# A new sharpshooter genus for *Sphinctogonia lingula* Yang & Li (Hemiptera, Cicadellidae, Cicadellini) from China

**DOI:** 10.3897/zookeys.561.6079

**Published:** 2016-02-08

**Authors:** Ze-hong Meng, Mao-fa Yang, Yu-feng Zhou

**Affiliations:** 1 Institute of Entomology, Guizhou University; Guizhou Provincial Key Laboratory for Agricultural Pest Management of the Mountainous Region, Guiyang, Guizhou, 550025, P. R. China; 2 Guizhou Tea Research Institute, Guizhou Academy of Agricultural Sciences, Guiyang, Guizhou, 550006, P. R. China; 3 College of Tobacco Science, Guizhou University, Guiyang, Guizhou, 550025, P. R. China

**Keywords:** Auchenorrhyncha, leafhopper, Cicadellinae, new combination, taxonomy

## Abstract

A new genus, *Sphinctogoniella*, is described to accommodate *Sphinctogonia lingula* Yang & Li, 2002, its type species from China. *Sphinctogoniella lingula* (Yang & Li, 2002), **comb. n.** is re-described and illustrated. Differences between the new genus and *Sphinctogonia* Breddin, 1901 are tabulated.

## Introduction

The Old World Cicadellinae genus *Sphinctogonia* Breddin, 1901 (type species: *Sphinctogonia lineolata* (Walker, 1857), Fig. 20) comprises currently nine species (Wilson et al. 2009) from Borneo, Laos, Malaysia, Indonesia and China (Young 1986; Zhang and Kuoh 1993; Yang and Li 2002).

The generic placement of *Sphinctogonia lingula* Yang & Li (2002) from China is re-accessed based on examination of its type series and more recently collected material. Its shorter body size and different coloration and male genitalia from other congeners indicate that it is unsatisfactory to keep *Sphinctogonia lingula* in *Sphinctogonia*. In addition, we have found that *Sphinctogonia lingula* cannot be classified into any known cicadelline genus. Thus, the purpose of this paper is to erect a new genus to accommodate it together with its redescription.

## Material and methods

The male and female genital structures were prepared according to the techniques described by Oman (1949) and Mejdalani (1998), respectively. The dissected parts are stored in small vials with glycerin and attached below the specimens. The morphological terminology adopted herein follows mainly Young (1986) and Dietrich (2005), except for the female genitalia (Nielson 1965; Davis 1975; Mejdalani 1998).

The type specimens and other specimens are deposited in the following institutions whose names are abbreviated in the text as follows:

GUGC
 Institute of Entomology, Guizhou University, Guiyang, China 


BMNH
 The Natural History Museum, London, UK 


FAFU
 Fujian Agriculture and Forestry University, Fuzhou, China 


## Results

### Sphinctogoniella gen. n.

Taxon classificationAnimaliaHemipteraCicadellidae

http://zoobank.org/5B4E6341-797A-4F8D-AD0D-FAAB2C46BF49

Figs 1–6 , 13–18 , 26–32

#### Type species.

*Sphinctogonia lingula* Yang & Li, 2002.

#### Diagnosis.

The new genus can be recognized by the following combination of features: (1) head anteriorly broadly rounded with ocelli located on imaginary line between anterior eye angles; (2) forewing with membrane distinct, veins obscure; (3) male pygofer without processes, surface with macrosetae near posterior margin; (4) subgenital plates slender, apex acute; (5) aedeagus slender, articulating sub-basally with unpaired paraphysis; (6) paraphysis with long spiniform processes; (7) style slender, extending posteriorly well beyond apex of connective, apex curved, hook-shaped; (8) female abdominal sternum VII well produced from ligulate base.

#### Description.

Length. 8.7–9.8 mm.

Coloration. Head and thorax dorsum and forewings orange-red to red, with black markings.

**Figures 1–12.**
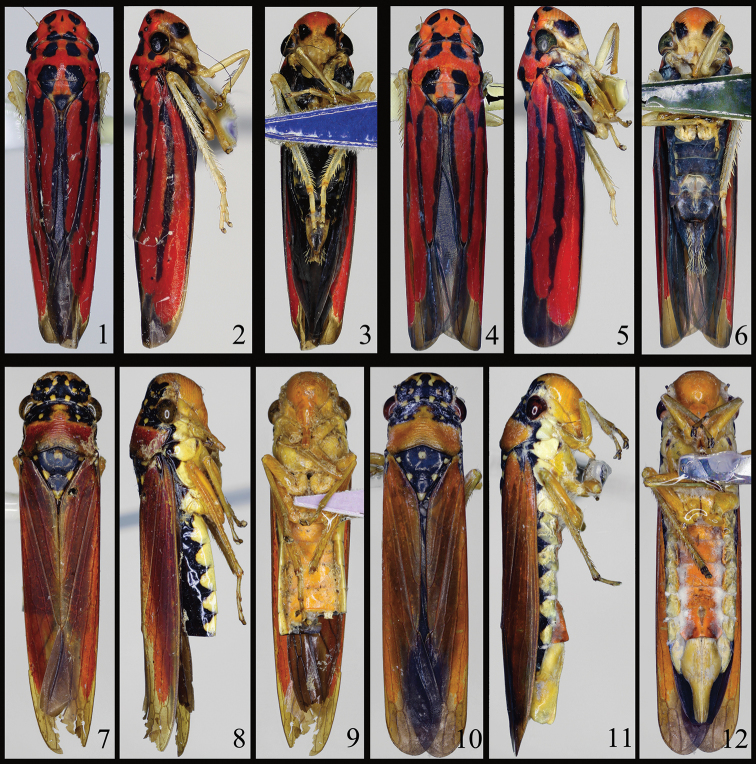
*Sphinctogoniella* and *Sphinctogonia* habitus. **1–6** *Sphinctogoniella lingula* (Yang & Li, 2002): **1–3** male (9.0 mm) **1** dorsal view **2** lateral view **3** ventral view **4–6** female (9.5 mm): **4** dorsal view **5** lateral view **6** ventral view **7–12** *Sphinctogonia lacta* Zhang & Kuoh, 1993: **7–9** male (15.9 mm) **7** dorsal view **8** lateral view **9** ventral view **10–12** female (16.7 mm) **10** dorsal view **11** lateral view **12** ventral view.

External features. Head (Figs 1–6) with anterior margin broadly rounded, median length slightly shorter than interocular width; crown with surface slightly convex, with fovea between ocelli and anterior angles of eyes; ocelli located on imaginary line between anterior eye angles, lateral frontal sutures extending onto crown, attaining ocelli; ocellus closer to adjacent eye than to each other; frontoclypeus flattened medially, muscle impressions distinct, anteclypeus convex longitudinally, apical margin sinuate, transclypeal suture indistinct medially. Pronotum (Figs 1, 4) slightly narrower than head, lateral margins divergent posteriorly, basal portion with transverse concavity, posterior margin slightly concave; mesonotum with surface of scutellum convex, transverse depression short and nearly straight; forewing with membrane distinct, veins obscure, base of second and third apical cells aligned transversely; hindleg with femoral setal formula 2:1:1.

**Figures 13–25.**
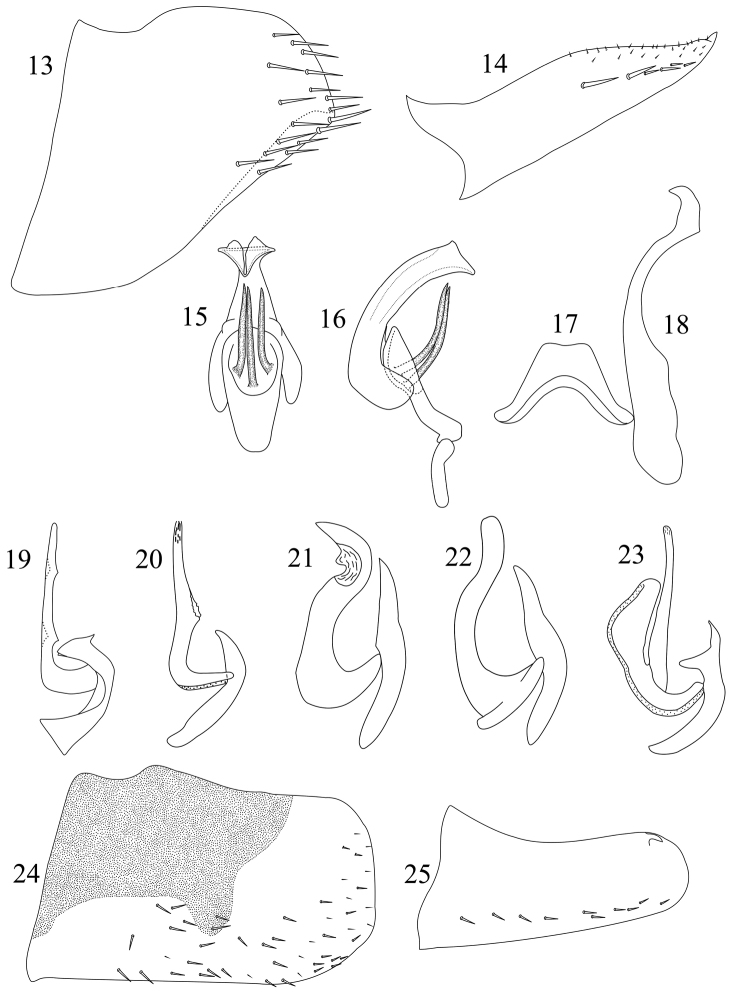
*Sphinctogoniella* and *Sphinctogonia* male genitalia. **13–18** *Sphinctogoniella lingula* (Yang & Li, 2002): **13** pygofer, lateral view **14** subgenital plate, ventral view **15** aedeagus and paraphysis, ventral view **16** connective, aedeagus and paraphysis, lateral view **17** connective, ventral view **18** style, ventral view **19–23** Lateral view of aedeagus and paraphysis: **19** *Sphinctogonia lacta* Zhang & Kuoh, 1993 **20** *Sphinctogonia lineolata* (Walker, 1857) **21** *Sphinctogonia comitatula* Melichar, 1926 **22** *Sphinctogonia servula* Breddin, 1901 **23** *Sphinctogonia avia* Young, 1986 **24–25** *Sphinctogonia lacta* Zhang & Kuoh, 1993: **24** pygofer, lateral view **25** subgenital plate, ventral view. **19–23** from Young (1986).

Male genitalia. Male pygofer lobes tapered to apex (Fig. 13), without processes, with macrosetae near posterior margin. Subgenital plates (Fig. 14) tapered to acute apex, distal half with uniseriate macrosetae medially and some short microsetae laterally. Aedeagus (Figs 15, 16) slender, articulating at its base with subapical part of paraphysis; gonopore apical on dorsal surface. Paraphysis (Figs 15, 16) unpaired with long spiniform processes. Connective (Fig. 17) broadly V-shaped. Style (Fig. 18) slender, extending posteriorly well beyond apex of connective, apex curved, hook-shaped.

**Figures 26–36.**
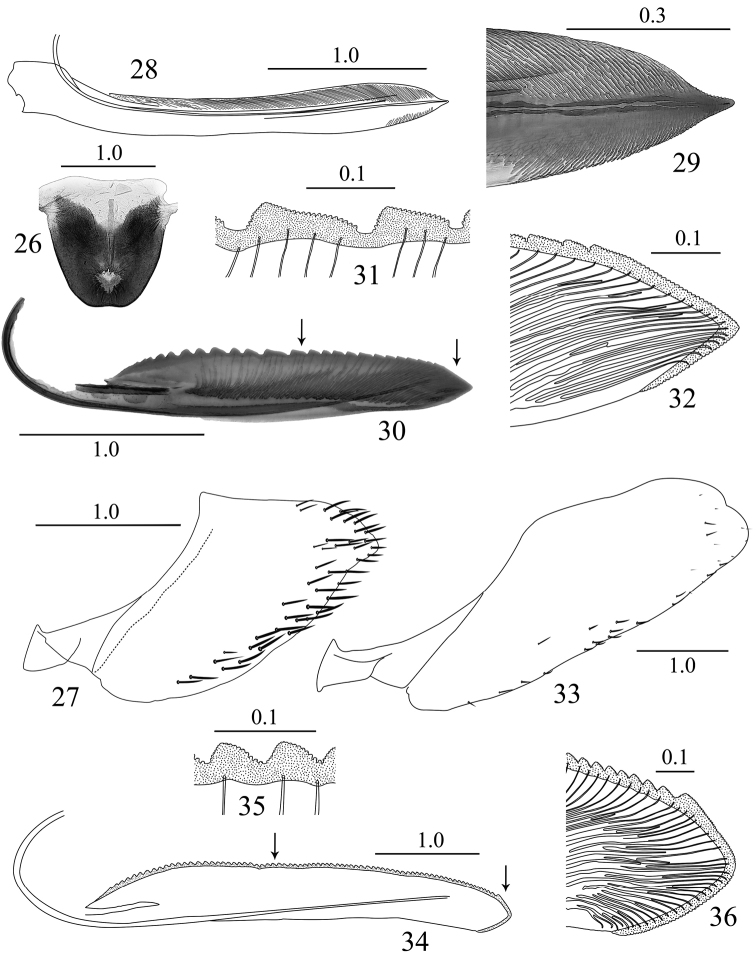
*Sphinctogoniella* and *Sphinctogonia* female genitalia. **26–32** *Sphinctogoniella lingula* (Yang & Li, 2002): **26** sternite VII, ventral view **27** pygofer, lateral view **28** valvula I, lateral view **29** apical portion of valvula I, lateral view **30** valvula II, lateral view **31** teeth of median portion of valvula II, lateral view **32** apical portion of valvula II, lateral view **33–36** *Sphinctogonia lacta* Zhang & Kuoh, 1993: **33** pygofer, lateral view **34** valvula II, lateral view **35** teeth of median portion of valvula II, lateral view **36** apical portion of valvula II, lateral view. Scale bars in millimeters.

Female genitalia. Sternite VII (Fig. 26) produced from ligulate base. Pygofer (Fig. 27), in lateral view, moderately produced; surface with macrosetae on posterior portion and ventral margin. Valvulae I (Figs 28, 29) of ovipositor, in lateral view, slightly expanded near apex; dorsal area with strigate sculpture in oblique lines extending from basal curvature to apex; ventral sculptured area restricted to apical portion, formed mostly by scale-like sculpture; apex of shaft acute. Valvulae II (Figs 30–32) of ovipositor, in lateral view, expanded beyond basal curvature; dorsal and ventral margins slightly convex; apex acute; preapical ventral prominence absent; 23 stout subtriangular teeth distributed from basal expanded portion to apical portion of shaft; teeth and apical portion of shaft bearing denticles. Gonoplacs, in lateral view, with basal half narrow and apical half distinctly expanded; apex rounded; surface with few setae on apical portion.

#### Distribution.

China (Zhejiang, Fujian, Guangxi, Guizhou).

#### Etymology.

The generic name is derived from *Sphinctogonia*. The gender of the genus is feminine.

#### Remarks.

In Young’s (1986) key to genera of the Old World Cicadellini the new genus runs to *Nanatka* Young, 1986, but differs from this genus in having a greater body size, the hindleg with femoral setal formula 2:1:1, the subgenital plates slender and acute apically (Fig. 14), and the paraphysis with long spiniform processes subapically (Figs 15, 16). Although the new genus also shares similarities with *Sphinctogonia* Breddin, 1901, such as the roundly produced head (Figs 1 and 4) and the male pygofer without processes (Fig. 13), it differs in the various features shown in Table 1.

**Table 1.** Differences between *Sphinctogoniella* **gen. n.** and *Sphinctogonia* Breddin, 1901.

	*Sphinctogoniella* gen. n.	*Sphinctogonia* Breddin, 1901
body size	8.7–9.8 mm	12.8–18.6 mm
pronotum	not constricted	clearly constricted
forewing	with distinct apical membrane, veins obscure	apical membrane absent, veins distinct
hindleg femoral setal formula	2:1:1	variable
male pygofer	slightly produced, with macrosetae on posterior area	moderately produced, with small weak setae on posterior and ventral area
subgenital plates	slender and acute apically; with uniseriate macrosetae medially and some short microsetae	often spine-like apically; with group of small weak setae, usually not arranged in rows
paraphysis	spoon-shaped in lateral view; with long spiniform processes subapically (Figs 15, 16)	usually boat-shaped in lateral view; without long processes, or with small teeth apically (Figs 19–23)
female pygofer	angular apically, with macrosetae on apical and ventral area (Fig. 27)	round apically, with small setae near apical and ventral area (Fig. 33)
valvula II	bearing less than 30 teeth on dorsal margin of blade (Fig. 30)	bearing many teeth (more than 50) on dorsal margin of blade (Fig. 34)

### Sphinctogoniella lingula

Taxon classificationAnimaliaHemipteraCicadellidae

(Yang & Li, 2002) comb. n.

Figs 1–6 , 13–18 , 26–32

Sphinctogonia lingula Yang & Li *in* Li & Jin, 2002: 176.

#### Description.

Length of males 8.7–9.5 mm, females 9.0–9.8 mm.

Coloration. Head and thorax dorsum and forewings orange-red to red, eyes and ocelli black. Head with two round black spots at apex; crown with anterior two black spots in front of ocelli, median portion with small black spot; basal margin with two connected triangular or trapeziform black spots behind ocelli. Pronotum with transverse anterior and posterior four black spots, anterior median two connecting with basal two black spots of crown, posterior median two connecting with black spots of basal angles of mesonotum; mesonotum with triangular black spots on basal angles, scutellum with large black spot; forewing with two longitudinal slender black stripes medially, basal angle black, apical membrane black brown, inner and outer margins black brown. Face orange yellow or off-white, apical portion of frontoclypeus with pair of lateral large black spots; anteclypeus with apico-median black marking in some specimens. Thoracic venter black brown to black, legs pale yellow brown. Abdominal venter black, sternites of posterior margin yellow white.

Male genitalia. Male pygofer lobes tapered to apex (Fig. 13) without process, with macrosetae near posterior margin. Subgenital plates (Fig. 14) tapered to acute apex, distal half with uniseriate macrosetae medially and some short microsetae laterally. Aedeagus (Figs 15, 16) slender, in lateral view curved ventrally, of similar width throughout length with apex truncate; in ventral view broad basally tapered to near apex then expanded apically with a pair of triangular flaps; articulating at its base with subapical part of paraphysis; gonopore apical on dorsal surface. Paraphysis (Figs 15, 16) un-paired, with ventral surface of distal half concave with three elongate spiniform processes, curved dorsally. Connective (Fig. 17) broadly V-shaped. Style (Fig. 18) slender, extending posteriorly well beyond apex of connective, apex curved, hook-shaped.

Female genitalia. Sternite VII (Fig. 26) ligulately produced, posterior margin with shallow concavity medially; internal sternite VIII membranous. Pygofer (Fig. 27), in lateral view, moderately produced; posterior margin with a subtriangular apical lobe; surface with macrosetae on posterior portion and extending anteriorly along ventral margin beyond its midlength. Valvifers I, in lateral view, nearly oval, bases slightly narrower. Valvifers II with small group of clustered setae near articulation point. Valvulae I (Figs 28, 29) of ovipositor, in lateral view, slightly expanded near apex; dorsal sculptured area extending from basal curvature to apex, broader near apex, formed by scale-like processes arranged in oblique lines; ventral sculptured area restricted to apical portion, formed mostly by scale-like processes; apex of shaft acute; dorsal margin forming denticles on apical portion of shaft. Valvulae II (Figs 30–32) of ovipositor, in lateral view, well expanded beyond basal curvature; dorsal and ventral margins slightly convex; apex acute; preapical prominence absent; approximately 23 teeth distributed from basal expanded portion to apical portion of shaft; all teeth subtriangular, basal teeth with superior angle slightly rounded, later with flat posterior area; median teeth strongly produced; apical portion with dentate dorsal margin longer than ventral margin; ducts extending toward teeth and toward apical portion of shaft. Gonoplacs, in lateral view, with basal half narrow and apical half distinctly expanded; apex rounded; surface with few setae on apical portion.

#### Distribution.

China (Zhejiang, Fujian, Guangxi, Guizhou).

#### Material examined.

1♂ (Holotype, FAFU), China, Fujian Province, Jianyang County, 7 April 1960, coll. Ma Cheng-lin; 1♀ (BMNH), China, Fujian Province, Yongan, Tianbaoshan, 17 May 2012, coll. Chang Zhi-min; 1♀ (GUGC), China, Fujian Province, Shilin County, 21 May 2012, coll. Long Jian-kun; 1♀ (Paratype, GUGC), China, Guangxi Province, Huaping, 5 June 1997, coll. Yang Mao-fa; 1♀ (GUGC), China, Guangxi Province, Huaping, 13 May 2014, coll. Qu Ling, Wu Yun-fei and Yang Hang; 1♂ (GUGC), China, Guangxi Province, Guilin, 26 April 2012, coll. Yang Zai-hua; 1♂ (BMNH), China, Guangxi Province, Guilin, 26 April 2012, coll. Zheng Wei-bin; 3♀♀ (Paratype, GUGC), China, Guizhou Province, Maolan, 26–30 May 1998, coll. Li Zi-zhong and Wang Lian-min; 3♂♂ (GUGC), 5♀♀ (GUGC), China, Guizhou Province, Daozhen County, Dashahe, 22–27 May 2004, coll. Song Qiong-zhang, Zhang Bin, Xu Fang-ling and Xu Pian; 1♀ (GUGC), China, Guizhou Province, Shibing County, Yuntaishan, 20 May 2009, coll. Yang Zai-hua.

## Supplementary Material

XML Treatment for Sphinctogoniella

XML Treatment for Sphinctogoniella lingula
